# Does the care dependency of nursing home residents influence their health-related quality of life?-A cross-sectional study

**DOI:** 10.1186/1477-7525-11-41

**Published:** 2013-03-11

**Authors:** Manuela Tabali, Thomas Ostermann, Elke Jeschke, Theo Dassen, Cornelia Heinze

**Affiliations:** 1 Department of Nursing Science, Charité- Universitätsmedizin Berlin, Augustenburgerplatz 1, Berlin, 13353, Germany; 2 Institute for Integrative Medicine, University of Witten/Herdecke, Gerhard-Kienle-Weg 4, Herdecke, 58313, Germany; 3 Scientific Institute of the medical insurance plan, Rosenthaler Straße 31, Berlin, 10178, Germany; 4 Protestant University of Applied Sciences Berlin, Teltower Damm 118, Berlin, 14167, Germany

**Keywords:** Heath-related quality of life, Care dependency, Nursing home residents

## Abstract

**Background:**

Studies on health-related quality of life (HRQOL) are missing for nursing home residents independent from their health conditions or interventions after admission. Our aim was to analyse if the care dependency of nursing home residents influence their HRQOL and to describe HRQOL of nursing home residents at the time of admission.

**Method:**

Eleven German nursing homes were randomly selected for a cross-sectional multicentre study from April 2008 until December 2009. HRQOL was measured with the Nottingham Health Profile (NHP) in the six domains “Physical Mobility”, ”Energy”, “Pain”, “Social Isolation”, “Emotional Reaction” and “Sleep”. Domain scores range from zero (good subjective health status) to 100 (poor subjective health status). Care dependency was evaluated using the Care Dependency Scale, age, sex, cognitive status and diseases were documented by the research assistants. Multivariate regression analysis was performed to quantify the influence of care dependency on HRQOL.

**Results:**

120 residents were included in total. HRQOL was mostly reduced in the domains “Physical Mobility” and ”Energy“ (mean scores >43.0), while impairment differences in the domains “Pain”, “Social Isolation”, “Emotional Reaction” and “Sleep” were only moderate (≤25.0). HRQOL was not influenced by the age. Women (n = 85) had a significantly poorer HRQOL in the domain “Pain” than men (mean score women: 29.5 ± 31.5; males: 14.9 ± 17.2; p = 0.011). Care dependency had an influence on the domain “Sleep” (ß = −0.195, p = 0.031), while the other domains were not influenced by care dependency. Residents with a low care dependency scored significantly lower (better HRQOL) in the domain “Sleep” than residents with a high care dependency (mean score 15.3; SD ± 19.0 versus mean score 32.8 SD ± 33.2; p < 0.02).

**Conclusion:**

The level of care dependency has no influence on the HRQOL from the nursing home residents’ perspective apart from the domain “Sleep”. High care dependency residents have a lower HRQOL in the domain “Sleep” compared to moderate and low care dependency residents. We found a significantly lower HRQOL in women compared to men in the domain “Pain“.

## Background

The average age of OECD country populations is increasing. As a consequence, the oldest population cohort grows fastest. The population aged 80 years and above has the most pronounced need for care, which is for example provided in nursing homes [1]. Nursing homes provide the majority of institutional care for elderly people with a need of care, which could not be met when living at home [2]. In Europe between two and ten percent of the elderly people live in nursing homes [3,4]. Within Europe, Germany is currently one of the four countries with the oldest population and is exceeded worldwide only by Japan [5]. In Germany four percent of the people aged 65 years and above live in nursing homes, with 49% of them being older than 85 years. In 2007 there were a total of 2.25 million care dependent persons, which was an increase of 6% compared to the year 2005 [6].

Care dependency has been described as: “The professional support to a patient whose self-care abilities have decreased and whose care demands make him/her to a certain degree dependent. The aim of the support is to restore the patient’s independency in performing self care” [7]. Care dependency residents are mostly in need of help with basic activities of daily living over an extended period of time, like bathing, dressing, eating and moving around. Thus, the needs of residents are due to long-term chronic and multimorbid conditions, which cause physical and/or mental disabilities and may influence their health-related quality of life (HRQOL) [8] and finally lead to a high degree of care dependency [9,10].

HRQOL has been defined as an individual’s subjective experience of the impact that illnesses and their treatments have on the individual’s functioning in a variety of domains such as physical, psychological and social functioning as well as the impact of illnesses on the ability to engage in activities of daily living [11]. HRQOL scores might therefore be interpreted as reflections of the resident’s own experience of gained (or lost) HRQOL and provide a non-disease specific outcome measure [12].

HRQOL is one of the most frequently used indicators to assess outcome for health care programs i.e. in rehabilitation medicine and in evaluating the health status of elderly people.

Only some studies have investigated the influence of care dependency and HRQOL without getting a clear picture. While Gonzales- Salvador et al. [13] found a significantly reduced quality of life in residents/patients with high care dependency, a study of Menzi-Kuhn [14] in Switzerland did not find a correlation between care dependency and HRQOL. In Germany, no study has investigated the influence of care dependency and HRQOL so far.

Therefore the following research questions were addressed in the current study:

1. Does the care dependency of nursing home residents influence their health-related quality of life of ?

2. What is the health-related quality of life of nursing home residents at the time of their admission?

## Methods

### Study design

The present study was designed as a cross-sectional study nested in the project “Health-related quality of life of residents in nursing homes in Germany”, which aims at evaluating the HRQOL with regard to pressure ulcer, falls, incontinence, care dependency and structural factors of the nursing homes such as staff qualification and activities provided for the residents. Ethical approval was given by the Ethical Committee of the Charité-University Medicine Berlin, (No: EA1/212/07).

### Setting and participants

Eleven nursing homes with a minimum of 50 beds out of 288 in Berlin and Brandenburg were randomly selected for this study. A total of 553 residents newly admitted to the nursing homes, or their legal advocates, were informed of the project. Inclusion criterion was the written informed consent given by the nursing home resident or the relevant legal advocate within the first two weeks upon nursing home admission. Residents were excluded if they were in a final stage of life with a survival probability of less than four weeks, in short-term care with a planned stay of less than four weeks and had a severe cognitive impairment.

### Questionnaire

Health-related quality of life was measured with the Nottingham Health Profile (NHP). The NHP was designed to be a standardized and simple measuring instrument of the subjective health status in the physical, social and emotional domains. It was validated in different settings and up to day translated into 22 languages [15]. It has proven its feasibility for nursing home residents with normal cognitive function and moderate cognitive impairment [16].

The German validated version of the NHP consists of 38 items in six domains; “Energy” (3 items), “Pain” (8 items), “Emotional Reactions” (9 items), “Sleep” (5 items), “Social Isolation” (5 items) and “Physical Mobility” (8 items). The items are answered by “yes” if the statement adequately reflected his /her current status or feeling or by “no” otherwise. Positive responses were weighted based on Thurstone’s Method of Paired Comparisons. Negative responses were not weighted. The values of all statements belonging to the domain were added up and thus produced the domain total. Domain scores range from zero (good subjective health status) to 100 (poor subjective health status) [17].

For our purpose and in agreement with the German author Kohlmann the NHP was modified to questions. For instance, the original statement “Everything is an effort.” in our study was changed into “Is everything an effort for you?”. The modified version was previously tested and showed acceptable results [18].

Care dependency was evaluated using the Care Dependency Scale (CDS), which is based on Henderson’s theory and consists of 15 items and includes various physical and psychosocial items (e.g. mobility, hygiene, nutrition, communication, sense of rules and values). The CDS fulfils the quality criteria for psychometric tests and has up to day been translated into 12 languages [10]. It is widely used in the field of geriatric and geronto-psychiatric research [19] and has been validated in long-term care populations [10]. Scores can range from 15 (high care dependency) to 75 (no care dependency). Score values can be categorized into “low care dependency” (60 to 75 points), moderate care dependency (45 to 59 points) and high care dependency (15 to 44 points) [20].

### Data collection

The recruitment period lasted from April 2008 until December 2009. The data collection was performed by trained research assistants. The modified NHP was collected in the second week upon nursing home admission in the resident’s room. Where the resident was unable to complete the NHP independently due to visual or other physical impairments, NHP interviews were conducted by research assistants. If the resident was unable to answer the questions, for example if he/she could not communicate with the research assistant or was disoriented and talked about other things than those asked, the NHP was not collected.

Care dependency of each resident was collected from the responsible nurse. The resident’s age, sex, cognitive status and diseases were documented by the research assistants using a machine-readable data form.

The cognitive status of each resident was evaluated using the Mini-Mental State Examination (MMSE) with scores ranging from 0 (very severe cognitive impairment) to 30 (no cognitive impairment). The MMSE is widely used and has been validated in long-term care populations, such as nursing homes [21,22].

Diseases of the residents were recorded from the patient’s file using the chapters of the International Classification of Diseases (ICD-10) [23].

All completed forms were scanned, evaluated, verified and exported to a database.

### Statistical analysis

Demographic variables were analyzed using descriptive statistics. Continuous variables were summarized using mean and standard deviation (SD). In the case of nominal variables comparisons between groups were made using the Chi-square Test. In case of continuous variables group differences were evaluated using t-test (for 2 groups) or one factorial analysis of variance (for more than two groups). Multivariate regression analysis was performed to quantify the influence of care dependency on HRQOL. Therefore, the six domains of the NHP were considered as dependent variables, while CDS scores together with age and gender were considered to be independent variables. Due to a low number of missing values we in accordance with Donner [24] decided to replace missing values of the CDS with mean values. To allow for comparisons between the six domains, the standardized regression coefficients ß and p-values were calculated. Autocorrelation was determined by Durbin-Watson statistics, whereby test-statistics between 1.5 and 2.5 indicate no serious autocorrelation. Model quality and accuracy was assessed using determination coefficient R^2^[25]. The complete statistical analysis was performed with IBM SPSS Statistics 19.0 for Windows.

## Results

### Sample

**Figure 1:**
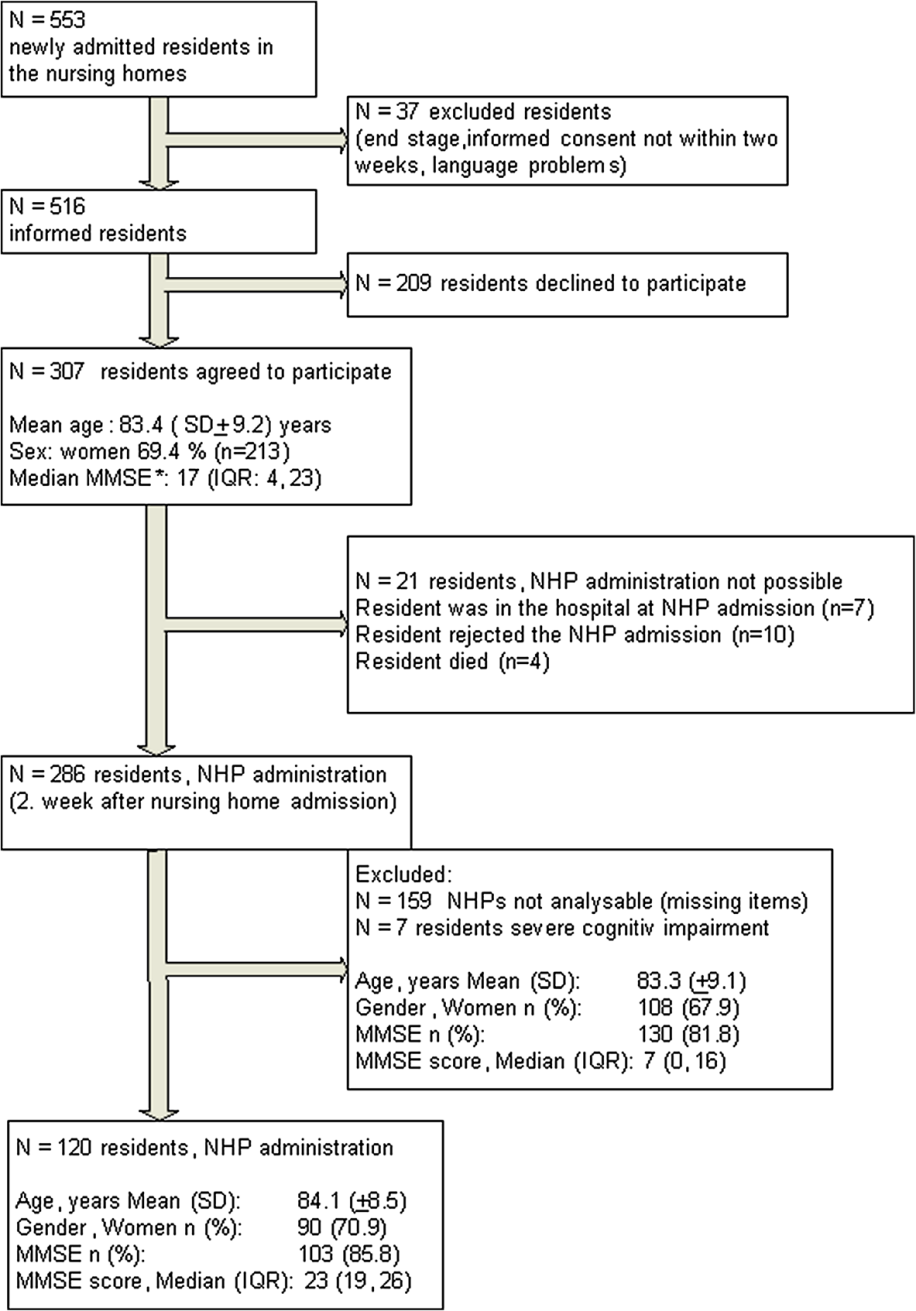
**Study population.** NHP = Nottingham Health Profile. MMSE = Mini-Mental State Examination.

From a total of 553 newly administered residents, 120 residents were included in the present analysis after checking for inclusion and exclusion criteria (Figure 1). The mean age of residents was 84.0 years (SD ± 8.4), with 45.0% (n = 54) of the study participants aged younger than 85 years. Most of the residents in our study were female (70.8%, n = 85). 12.5% (n = 15) were single, 65.0% (n = 78) were widowed and 22.5% (n = 27) were married or were in a steady relationship with a partner.

Prior to nursing home admission 43.3% (n = 52) of the residents had lived at home, 45.8% (n = 55) had been in hospitals or in long-term care institutions and 10.9% (n = 13) in other situations. Most of the residents were suffering from diseases of the circulatory system (n = 98; 81.7%), followed by mental and behavioural disorders (n = 69; 57.5%), endocrine, nutritional and metabolic diseases (n = 61; 50.8%), diseases of the musculoskeletal system and connective tissue(n = 57; 47.5%), and diseases of the genitourinary system (n = 53; 44.2%). Eighty-two percent of the residents (n = 99) were diagnosed with three or more diseases.

Care dependency was determined in 92.5% (n = 111) of the residents with a mean of 53.9 (SD ± 11.9) in the CDS. The group of residents in which care dependency values were missing (n = 9) did not differ with regard to age (84.2 years; SD ± 8.6; p = 0.327) and gender (women n = 7; p = 0.634) from the group of residents in which care dependency was determined.

A total of 30.8% (n = 37) had a low care dependency, 42.5% (n = 51) had a moderate care dependency and 19.2% (n = 23) had a high care dependency.

### Health-related quality of life

HRQOL was measured in the domains “Energy”, “Pain”, “Emotional Reactions”, “Sleep”, “Social Isolation” and “Physical Mobility” with domain scores ranging from zero (good subjective health status) to 100 (poor subjective health status).

The domain “Emotional Reaction” had the lowest mean scores of all the NHP domains with 19.2 (SD ± 22.7). The questions most frequently answered with “yes” in this domain were “Things are getting me down” (40.0%; n = 48). 30.8% (n = 37) answered all nine questions about “Emotional Reaction” with “no”. Only one resident answered all questions with “yes”.

The second lowest score of the NHP was noticed in the domain “Sleep” (mean 22.0; SD ± 27.0), with “I‘ m waking up in the early hours of the morning” being most frequently answered with “yes” (40.0%; n = 48). Overall, 60.8 (n = 73) of the residents answered at least one out of five sleep questions with yes. Three residents answered all question with “yes”.

The domain “Social Isolation” is placed on the third position of all NHP domains with a mean score of 22.4 (SD ± 26.3). The question most frequently answered with “yes” was “I feel lonely” (36.7%; n = 44). 39.2% (n = 47) of the residents had no problems with “Social Isolation” and answered all question in this domain with “no”. Three residents answered all questions with “yes”.

The domain “Pain” followed on the fourth position of the NHP domains with a mean score of 25.2 ± 28.8. The questions in this domain that the residents most frequently answered with “yes” were ”I’m in pain when I walk” and “I’m in pain when climbing up and down stairs or steps” (42.5%; n = 51). Thirty-two residents (25.8%; n = 31) stated not to be in pain at all and replied to all eight questions about pain with “no”. Four residents answered all eight questions about pain with “yes”.

The domain “Energy” had a mean score of 43.7 ± 37.1. The question most frequently answered with “yes” was ”I soon run out of energy” (52.5%; n = 63). 30.0% (n = 36) had no problems with their “Energy”. 20.0% (n = 24) answered all three questions about their “Energy” with “yes”.

The highest score was discovered in the domain “Physical Mobility” with 53.5 (SD ± 24.0). Over 50.0% of the residents answered five out of eight questions in this domain with “yes”, i.e. “I need help to walk about outside” was answered with “yes” by 101 residents (84.2%). Four residents had no problems at all with their “Physical Mobility” and answered all eight questions with “no”, five residents answered all questions in this domain with “yes”. An overview of all 38 questions answered with “yes” is given in Table 1.

**Table 1:** Frequency of all 38 items of the Nottingham health profile that residents answered with “yes” (N = 120)

**Domain**	**Item**	**Yes n (%)**	**Missing n (%)**
Energy			
	I´m tired all the time	41 (34.2)	0
	**I soon run out of energy**	**63 (52.5)**	0
	Everything is a strenuous effort	58 (48.3)	0
Pain			
	I have pain at night	24 (20.0)	0
	I find it painful to change position	34 (28.3)	1 (0.8)
	I have unbearable pain	13 (10.8)	0
	**I’m in pain when I walk**	**51 (42.5)**	0
	I´m in pain when I´m standing	46 (38.3)	0
	I´m in pain when I´m sitting	29 (24.2)	2 (1.7)
	I’m constant pain	26 (21.7)	0
	**I’m in pain when going up and down staires or steps**	**51 (42.5)**	22 (18.3)
Emotional reaction			
	**Things are getting me down**	**48 (40.0)**	0
	I´ve forgotten what it´s like to enjoy myself	24 (20.0)	1 (0.8)
	I´m feeling on edge	10 (8.3)	0
	The day seem to drag	47 (39.2)	0
	I lose my temper easily these days	17 (14.2)	0
	I feel as if I´m losing control	7 (5.8)	1 (0.8)
	Worry is keeping me awake at night	19 (15.8)	2 (1.7)
	I feel life is not worth living	25 (20.8)	4 (3.3)
	I wake up feeling depressed	20 (16.7)	0
Sleep			
	I take tablets to help me sleep	12 (10.0)	0
	I lie awake for most of the night	22 (18.3)	0
	**I’m waking up in the early hours of the morning**	**48 (40.0)**	0
	I sleep badly at night	31 (25.8)	3 (2.5)
	It takes me long time to get to sleep	31 (25.8)	0
Social isolation			
	**I feel lonely**	**44 (36.7)**	1 (0.8)
	I´m finding it hard to made contact with people	33 (27.5)	1 (0.8)
	I feel there is nobody I am close to	15 (12.5)	1 (0.8)
	I finding it hard to get on with people	15 (12.5)	1 (0.8)
	I feel I am a burden to people	24 (20.0)	3 (2.5)
Physical mobility			
	I can only walk about indoors	52 (43.3)	0
	I find it hard to bend	80 (66.7)	0
	I´m unable to walk all	8 (6.7)	1 (0.8)
	I have trouble getting up and down stairs or steps	91 (75.8)	2 (1.7)
	I find it hard to reach for things	59 (49.2)	1 (0.8)
	I find it hard to dress myself	71 (59.2)	2 (1.7)
	I find it hard to stand for long	98 (81.7)	1 (0.8)
	**I need help to walk about outside**	**101 (84.2)**	1 (0.8)

### Univariate analysis

The values of all HRQOL domains were not influenced by age (<85 years vs. ≥ 85 years; p > 0.05 in all domains). With respect to gender, we found a difference in the domain “Pain”, where, compared to male residents, women (n = 85) had a significantly higher score (poorer HRQOL) (mean score women: 29.5 ± 31.5; males: 14.9 ± 17.2; p = 0.011) (Table 2).

**Table 2:** Health-related quality of life measured with the Nottingham health profile according to the care dependency, gender and age of residents

	**Energy**	**Pain**	**Emotional reaction**	**Sleep**	**Social isolation**	**Physical mobility**
	**Mean (SD)**	**Mean (SD)**	**Mean (SD)**	**Mean (SD)**	**Mean (SD)**	**Mean (SD)**
**Care dependency**						
Low care dependency (n = 37)	42.6 (39.2)	29.6 (32.4)	15.7 (22.5)	15.3 (19.0)	19.7 (20.8)	52.0 (19.3)
Moderate care dependency (n = 51)	39.3 (33.9)	21.2 (21.7)	18.8 (20.1)	20.4 (24.2)	21.7 (25.5)	54.3 (24.7)
High care dependency (n = 23)	49.5 (40.5)	27.1 (33.9)	23.6 (26.8)	32.8 (33.2)	22.9 (33.5)	57.1 (25.3)
p-value (ANOVA)	0.549	0.367	0.418	0.030	0.883	0.712
**Age**						
<85 years (n = 54)	49.2 (41.0)	25.4 (28.7)	19.3 (22.3)	24.7 (29.5)	22.8 (28.6)	52.7 (24.5)
≥85 years (n = 66)	39.2 (33.2)	25.2 (29.2)	19.1 (23.2)	19.8 (24.7)	22.1 (24.4)	54.2 (23.8)
p-value (T-Test)	0.143	0.961	0.966	0.325	0.877	0.749
**Gender**						
Female (n = 85)	47.5 (37.3)	29.5 (31.5)	20.2 (24.2)	25.0 (29.3)	20.1 (25.1)	53.7 (25.3)
Male (n = 35)	34.9 (35.6)	14.9 (17.2)	16.8 (18.9)	14.7 (12.6)	28.0 (28.5)	52.9 (20.8)
p-value (T-Test)	0.091	0.011	0.456	0.057	0.137	0.867

**Figure 2:**
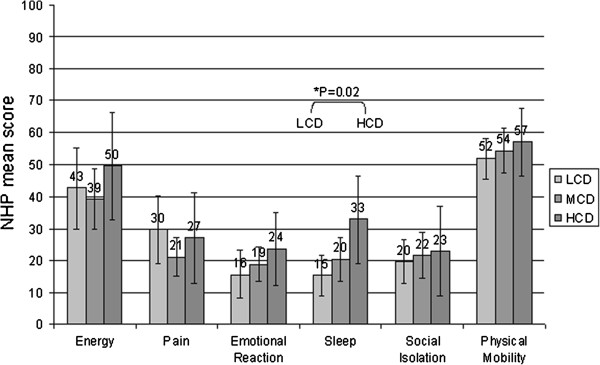
**Care dependency Level according to Health related quality of life measured with the Nottingham Health Profile (NHP).** LCD = Low care dependency (N = 37). MCD = Moderate care dependency (N = 51). HCD = High care dependency (N = 23).

Independent from the care dependency grade the highest score was found in the domain “Physical Mobility”, followed by the domain “Energy”. Residents with a low care dependency scored significantly lower (better HRQOL) in the domain “Sleep” than residents with a high care dependency (mean score 15.3; SD ± 19.0 versus mean score 32.8 SD ± 33.2; p < 0.02). No other significance was detected (Figure 2).

### Multivariate regression analyses

Table 3 summarizes the results of the regression analyses in terms of the standardized regression coefficients ß and p-values. For all six NHP domains regression analysis did not find any influence by age (regression coefficients ß between −0.017 for the domain “Pain” and 0.104 for the domain “Physical Mobility”) with the exception of the domain “Pain”, which was influenced by gender (ß = −0.238, p = 0.015). HRQOL was not influenced by gender either. Care dependency had an impact on the domain “Sleep” (ß = −0.195, p = 0.031), while the other domains were not affected by care dependency. Linear regression analysis therefore confirms the results of unvaried statistics.

**Table 3:** Multivariate regression analysis according to the influence of care dependency, age and gender on health-related quality of life measured with the Nottingham health profile

	**Care dependency score**	**Age**	**Gender**
**Energy**			
	Durbin Watson: 0.1691 R Quadrat: 0,030		
Beta [95% CI]	−0.075 [−0.835, 0.344]	0.005 [−0.830, 0.872]	−0.152 [−28.041, 3,379]
T	−0.825	0.049	−1.555
Sig.	0.411	0.961	0.123
**Pain**			
	Durbin Watson:1.798 R Quadrat: 0,055		
Beta [95% CI]	0.029 [−0.379, 0.525]	−0.017 [−0.710, 0.596]	−0.238 [−27.074, -2.967]
T	0.321	−0.173	−2.468
Sig.	0.749	0.863	0.015
**Emotional reaction**			
	Durbin Watson:1.141 R Quadrat: 0,026		
Beta [95% CI]	−0.116 [0.592, 0.132]	0.094 [−0.270, 0.775]	−0.034 [−11.317, 7.980]
T	−1.260	0.957	−3.343
Sig.	0.210	0.341	0.733
**Sleep**			
	Durbin Watson:2.089 R Quadrat: 0,069		
Beta [95% CI]	−0.195 [−0.882, -0.042]	−0.026 [−0.689, 0.524]	−0.178 [−21.765, 0.624]
T	−2.179	−0.2269	−1.870
Sig.	0.031	0.788	0.064
**Social isolation**			
	Durbin Watson:1.556 R Quadrat: 0,028		
Beta [95% CI]	−0.056 [−0.546, 0.28]	0.087 [−0.332,, 0.875]	0.168 [−1.465, 20.806]
T	−0.609	0.892	1.720
Sig.	0.544	0.374	0.088
**Physical mobility**			
	Durbin Watson:1.745 R Quadrat: 0,028		
Beta [95% CI]	−0.137 [−0.670, 0.094]	0.104 [−0.254, 0.850]	0.024 [−8.918, 11.442]
T	−1.492	1.070	0.246
Sig.	0.139	0.287	0.806

In all regression models except for “Energy” and “Emotional Reaction”, Durbin-Watson statistics were in an acceptable range between 1.6 (“Social Isolation”) and 2.09 (“Sleep”), However the explanatory power of the regression models is weak: Only between 2% and 7% of the variation in the domains of HRQOL can be explained by age, gender and CDS. For this reason results should be interpreted with caution.

## Discussion

This study presents results from eleven nursing homes in Germany about HRQOL from the resident’s perspective, which according to Kane et al. denotes the “gold standard” [26]. Measuring HRQOL provides an outstanding insight towards approaches that may lead to an improved quality of care [27]. HRQOL was measured in six dimension of the NHP.

As a main result of our study we found that care dependency of the nursing home residents does not influence HRQOL of the residents, except for the domain “Sleep”. Here, residents with a high care dependency, compared to those with a low care dependency, had a significantly lower HRQOL (mean score: high care dependency 32.8 vs. low care dependency 15.3, p = 0.02). This can be explained by the fact that high care dependency residents are in more need of care compared to other residents. Nurses, for example, have to check the residents during night for incontinence problems or to change positions to avoid pressures ulcer, which results in disturbed sleep.

All other HRQOL domains (“Energy”, “Pain”, Emotional Reaction”, Social Isolation” and “Physical Mobility”) both in the univariate and in the regression analysis showed no significant results in relation to care dependency. Therefore HRQOL in our setting does not seem to be influenced by the degree of care dependency which is comparable with the other studies in similar contexts [28,29]. It is worthy of note that the evaluation of HRQOL from the perspective of relatives, nurses or physicians does not necessary agree with the residents’ perspective [28-30], which shows the importance of this specific feature. This is particularly important for elder people and their perception of getting older in a permanently developing environment. Thus, standards and values in this group of people are of great importance for the assessment of HRQOL [31]. Moreover, aspects of a ”good“ or “normal” HRQOL might therefore be subject to change in the various periods of elderly people’s life compared to other patient groups [32].

Taking this into account the reasonable HRQOL in our setting is understandable. Almost all HRQOL scores were below fifty from a possible 100, the maximum was not reached. A literature search revealed results of adults discharged from hospitals to their own homes in Switzerland who had an even lower HRQOL in the domains “Emotional Reaction” (79.8) and “Social Isolation” (87.2) [33] compared to our results (“Emotional Reaction” mean score: 19.2 and “Social Isolation” mean score: 22.4 ). Another study on hip fracture patients in all six domains of the NHP showed a lower HRQOL than our setting one week after hospital admission. [34]. This could be explained by the aforementioned aging process and also the process of moving into a nursing home which is usually not based upon an abrupt decision but rather as a result of severe and acute problems associated with a hospital admission.

Becoming aware of an increasing age goes alongside with cognitive and emotional adaptation processes resulting in a recalibration of individual goals and beliefs. In addition to internal standards and values, conceptualization and reconsidering of quality of life is also a part of the adaptation process [35,36]. As a consequence, HRQOL in our setting can be better compared to studies in acute settings.

The highest reduction of HRQOL was seen in the domains “Physical Mobility” (mean score 53.5) indicating clearly the residents’ physical limitations. This is of course not surprising, as most nursing home residents prior to nursing home admission suffer from several chronic diseases leading to substantial physical dependency, which then becomes a predictor for admission to a nursing home [37,38]. However, the efforts made by residents to remain mobile use a lot of their energy, which is clearly reflected in the mean score of the domain “Energy” (43.7). In our study, more than fifty-two percent of the residents state that they “run out of energy”. One measure to improve this situation is to adapt exercise programmes which have already proven their effectiveness in other studies [39,40].

HRQOL was independent from the care dependency in the domains “Pain”, “Social Isolation”, “Emotional Reaction”, and “Sleep” with all mean scores being less than twenty five points. With respect to gender differences we only found a significantly lower HRQOL in women compared to men in the domain “Pain” (mean score 29.5 vs. 14.9, p = 0.011), which is confirmed by other studies [41,42]. An explanation might be that individuals who consider themselves more masculine and less sensitive to pain show higher pain thresholds and tolerances [42].

### Limitation

Out of all residents (n = 553) agreeing to participate only 41.4% could be included in the NHP analysis, which has already been discussed in detail [18]. In addition to the sample size of our study it cannot be regarded as representative. This is mainly due to the fact that the drop out was not by random but by systematic conditions (residents declined to participate, data administration not possible, cognitive impairment). Moreover the sample was drawn in Berlin and Brandenburg (only two out of sixteen federal German states), so it can’t be representative for nursing home residents “in general”. Finally the residents were surveyed at the time of admission which also might have influenced their response. For the survey we chose the instrument NHP owing to the dichotomized response scale with a completion time of about 10 minutes. The high number of missing answers in the item “I’m in pain when going up and down stairs or steps” (n = 22, 18.3%) can be attributed to the use of elevators, to living on ground floors or to not being applicable to the resident’s situation.

## Conclusion

Overall, nursing home residents regard their own HRQOL as reasonable. Our study showed that the level of care dependency has no influence on the HRQOL from the nursing home residents’ perspective except for the domain “Sleep”. High care dependency residents have a lower HRQOL in this domain compared to moderate and low care dependency residents. It is still necessary to evaluate whether activities of nursing staff for high care dependency residents might be structured in such a way that exhaustion due to sleep disturbance is avoided. We found a significantly lower HRQOL in women compared to men in the domain “Pain“. Gender aspects, too, should be examined in further studies. Most importantly the impact of aging processes in HRQOL should be made the subject of researches in a society that is growing older and older.

## Abbreviations

OECD: Organisation for Economic Co-operation and Development; HRQOL: Health-related quality of life; NHP: Nottingham health profile; MMSE: Mini-Mental State Examination; CDS: Care Dependency Scale; LCD: Low care dependency; MCD: Moderate care dependency; HCD: High care dependency.

## Competing interests

The authors declare that they have no competing interests. The research was not supported.

There is no financial relationship between the authors and any organisation.

## Authors’ contributions

MT participated in the design of the study and the performance of the statistical analysis and also drafted the manuscript. TO helped with the interpretation of the data, drafting or critical revision of the manuscript. EJ made substantial contributions to the interpretation of data and statistical analysis. TD agreed to the study and participated in its design. CH to approve of the study, participated in its design, coordination and critical revision of the manuscript. All authors have given final approval of the version to be published. All authors read and approved the final manuscript.
